# 2-(3-Ethyl­sulfanyl-5-fluoro-1-benzofuran-2-yl)acetic acid

**DOI:** 10.1107/S160053680903671X

**Published:** 2009-09-16

**Authors:** Hong Dae Choi, Pil Ja Seo, Byeng Wha Son, Uk Lee

**Affiliations:** a Department of Chemistry, Dongeui University, San 24 Kaya-dong Busanjin-gu, Busan 614-714, Republic of Korea; b Department of Chemistry, Pukyong National University, 599-1 Daeyeon 3-dong, Nam-gu, Busan 608-737, Republic of Korea

## Abstract

The title compound, C_12_H_11_FO_3_S, was prepared by alkaline hydrolysis of ethyl 2–(3–ethyl­sulfan­yl–5–fluoro–1–benzofuran–2–­yl) acetate. In the crystal structure, the carboxyl groups are involved in inter­molecular O—H⋯O hydrogen bonds, which link the mol­ecules into centrosymmetric dimers. These dimers are further packed into stacks along the *b* axis by aromatic π–π inter­actions between the furan ring and the benzene ring of neighbouring benzofuran ring systems [centroid–centroid distance = 3.684 (5) Å].

## Related literature

For the crystal structures of similar 2–(5–halo–1–benzofuran–2–­yl) acetic acid derivatives, see: Choi *et al.* (2009*a* ▶,*b* ▶). For the pharmacological properties of benzofuran compounds, see: Howlett *et al.* (1999 ▶); Twyman & Allsop (1999 ▶).
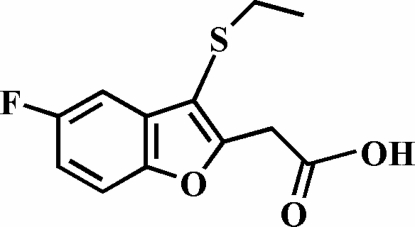


## Experimental

### 

#### Crystal data

C_12_H_11_FO_3_S
*M* *_r_* = 254.27
Monoclinic, 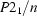

*a* = 10.6009 (9) Å
*b* = 8.3319 (7) Å
*c* = 13.395 (1) Å
β = 96.138 (1)°
*V* = 1176.34 (17) Å^3^
*Z* = 4
Mo *K*α radiation
μ = 0.28 mm^−1^
*T* = 173 K
0.25 × 0.20 × 0.16 mm

#### Data collection

Bruker SMART CCD diffractometer
Absorption correction: multi-scan (*SADABS*; Sheldrick, 2000 ▶) *T*_min_ = 0.931, *T*_max_ = 0.958
9646 measured reflections
2543 independent reflections
1541 reflections with *I* > 2σ(*I*)
*R*_int_ = 0.072

#### Refinement

*R*[*F* ^2^ > 2σ(*F* ^2^)] = 0.047
*wR*(*F* ^2^) = 0.113
*S* = 1.16
2543 reflections
159 parameters
H atoms treated by a mixture of independent and constrained refinement
Δρ_max_ = 0.28 e Å^−3^
Δρ_min_ = −0.33 e Å^−3^

### 

Data collection: *SMART* (Bruker, 2001 ▶); cell refinement: *SAINT* (Bruker, 2001 ▶); data reduction: *SAINT*; program(s) used to solve structure: *SHELXS97* (Sheldrick, 2008 ▶); program(s) used to refine structure: *SHELXL97* (Sheldrick, 2008 ▶); molecular graphics: *ORTEP-3* (Farrugia, 1997 ▶) and *DIAMOND* (Brandenburg, 1998 ▶); software used to prepare material for publication: *SHELXL97*.

## Supplementary Material

Crystal structure: contains datablocks I. DOI: 10.1107/S160053680903671X/zq2007sup1.cif

Structure factors: contains datablocks I. DOI: 10.1107/S160053680903671X/zq2007Isup2.hkl

Additional supplementary materials:  crystallographic information; 3D view; checkCIF report

## Figures and Tables

**Table 1:** Hydrogen-bond geometry (Å, °)

*D*—H⋯*A*	*D*—H	H⋯*A*	*D*⋯*A*	*D*—H⋯*A*
O2—H2⋯O3^i^	0.85 (5)	1.81 (5)	2.654 (3)	177 (5)

Symmetry code: (i) 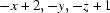.

**Fig. 1.**
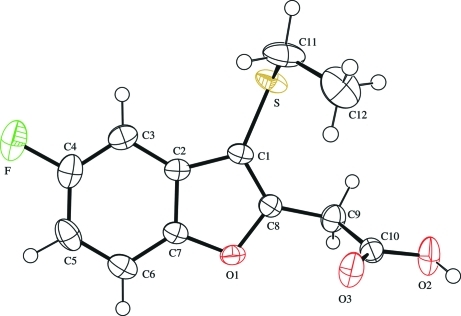
The molecular structure of the title compound with the atom numbering scheme. Displacement ellipsoids are drawn at the 50% probability level. H atoms are presented as small cycles of arbitrary radius.

**Fig. 2.**
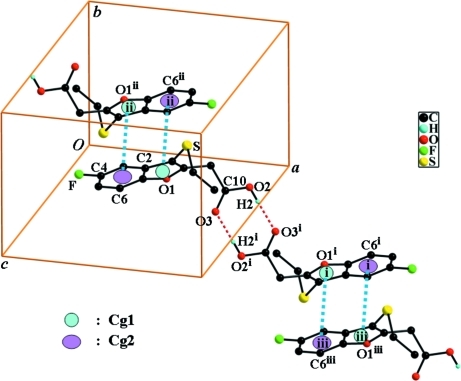
O—H···O and π···π interactions (dotted lines) in the title compound. Cg denotes the ring centroids. [Symmetry codes: (i) - x + 2, - y, - z + 1; (ii) - x + 1, - y + 1, - z + 1; (iii) x + 1, y - 1, z.]

## supplementary crystallographic information

### Comment

Molecules involving benzofuran moiety have attracted considerable interest in the view of their pharmacological properties (Howlett *et al.*, 1999; Twyman & Allsop, 1999). As a part of our ongoing studies on the synthesis and structures of 2–(5–halo–1–benzofuran–2–yl) acetic acid analogues, the crystal structures of 2–(5–bromo–3–methylsulfanyl–1–benzofuran–2–yl) acetic acid (Choi *et al.*, 2009*a*) and 2–(5–fluoro–3–methylsulfanyl–1–benzofuran–2–yl) acetic acid (Choi *et al.*, 2009*b*) have been described in the literature. Here we report the crystal structure of the title compound (Fig. 1).

The benzofuran unit is essentially planar, with a mean deviation of 0.005 (2) Å from the least-squares plane defined by the nine constituent atoms. In the crystal structure, the carboxyl groups are involved in intermolecular O—H···O hydrogen bonds (Table 1 and Fig. 2), which link the molecules into centrosymmetric dimers. These dimers are further packed into stacks along the *b* axis by aromatic π···π interactions between the furan ring and the benzene ring of adjacent benzofuran ring systems. The Cg1···Cg2^ii^ distance is 3.684 (5) Å (Fig. 2; Cg1 and Cg2 is the centroids of the C1/C2/C7/O1/C8 furan ring and the C2–C7 benzene ring, respectively).

### Experimental

Ethyl 2–(3–ethylsulfanyl–5–fluoro–1–benzofuran–2–yl) acetate (254 mg, 1.0 mmol) was added to a solution of potassium hydroxide (348 mg, 6.0 mmol) in water (20 ml) and methanol (20 ml), and the mixture was refluxed for 6h, then cooled. Water was added, and the solution was extracted with dichloromethane. The aqueous layer was acidified to pH 1 with concentrated hydrochloric acid and then extracted with chloroform, dried over magnesium sulfate, filtered and concentrated under vacuum. The residue was purified by column chromatography (ethyl acetate) to afford the title compound as a colorless solid [yield 87%, m.p. 401–402 K; *R*_f_ = 0.69 (ethyl acetate)]. Single crystals suitable for X–ray diffraction were prepared by evaporation of a solution of the title compound in benzene at room temperature. Spectroscopic analysis: EI–MS 254 [M^+^].

### Refinement

Atom H2 of the hydroxy group was found in a difference Fourier map and refined freely. The other H atoms were positioned geometrically and refined using a riding model, with C—H = 0.93 Å for the aryl, 0.97 Å for the methylene, and 0.96 Å for the methyl H atoms with *U*_iso_(H) = 1.2*U*_eq_(C) for the aryl and methylene H atoms, and 1.5*U*_eq_(C) for the methyl H atoms.

### Crystal data

**Table:**

C_12_H_11_FO_3_S	*F*(000) = 528
*M**_r_* = 254.27	*D*_x_ = 1.436 Mg m^−^^3^
Monoclinic, *P*2_1_/*n*	Mo *K*α radiation, λ = 0.71073 Å
Hall symbol: -P 2yn	Cell parameters from 2478 reflections
*a* = 10.6009 (9) Å	θ = 2.3–27.4°
*b* = 8.3319 (7) Å	µ = 0.28 mm^−^^1^
*c* = 13.395 (1) Å	*T* = 173 K
β = 96.138 (1)°	Block, colorless
*V* = 1176.34 (17) Å^3^	0.25 × 0.20 × 0.16 mm
*Z* = 4	

### Data collection

**Table:**

Bruker SMART CCD diffractometer	2543 independent reflections
Radiation source: fine-focus sealed tube	1541 reflections with *I* > 2σ(*I*)
graphite	*R*_int_ = 0.072
Detector resolution: 10.0 pixels mm^-1^	θ_max_ = 27.0°, θ_min_ = 2.3°
φ and ω scans	*h* = −13→13
Absorption correction: multi-scan (*SADABS*; Sheldrick, 2000)	*k* = −10→10
*T*_min_ = 0.931, *T*_max_ = 0.958	*l* = −17→17
9646 measured reflections	

### Refinement

**Table:**

Refinement on *F*^2^	Primary atom site location: structure-invariant direct methods
Least-squares matrix: full	Secondary atom site location: difference Fourier map
*R*[*F*^2^ > 2σ(*F*^2^)] = 0.047	Hydrogen site location: difference Fourier map
*wR*(*F*^2^) = 0.113	H atoms treated by a mixture of independent and constrained refinement
*S* = 1.16	*w* = 1/[σ^2^(*F*_o_^2^) + (0.*P*)^2^ + 1.581*P*] where *P* = (*F*_o_^2^ + 2*F*_c_^2^)/3
2543 reflections	(Δ/σ)_max_ < 0.001
159 parameters	Δρ_max_ = 0.28 e Å^−^^3^
0 restraints	Δρ_min_ = −0.33 e Å^−^^3^

### Special details

**Table:**

Geometry. All esds (except the esd in the dihedral angle between two l.s. planes) are estimated using the full covariance matrix. The cell esds are taken into account individually in the estimation of esds in distances, angles and torsion angles; correlations between esds in cell parameters are only used when they are defined by crystal symmetry. An approximate (isotropic) treatment of cell esds is used for estimating esds involving l.s. planes.
Refinement. Refinement of F^2^ against ALL reflections. The weighted R-factor wR and goodness of fit S are based on F^2^, conventional R-factors R are based on F, with F set to zero for negative F^2^. The threshold expression of F^2^ > 2sigma(F^2^) is used only for calculating R-factors(gt) etc. and is not relevant to the choice of reflections for refinement. R-factors based on F^2^ are statistically about twice as large as those based on F, and R- factors based on ALL data will be even larger.

### Fractional atomic coordinates and isotropic or equivalent isotropic displacement parameters (Å^2^)

**Table:**

	*x*	*y*	*z*	*U*_iso_*/*U*_eq_	
S	0.76702 (8)	0.59687 (10)	0.63171 (7)	0.0380 (2)	
F	0.2776 (2)	0.4175 (3)	0.75176 (16)	0.0561 (6)	
O1	0.5990 (2)	0.2294 (2)	0.48565 (15)	0.0279 (5)	
O2	0.9881 (2)	0.1796 (3)	0.4247 (2)	0.0459 (7)	
H2	1.034 (5)	0.097 (6)	0.437 (4)	0.11 (2)*	
O3	0.8642 (2)	0.0735 (3)	0.53184 (18)	0.0403 (6)	
C1	0.6717 (3)	0.4365 (3)	0.5848 (2)	0.0252 (7)	
C2	0.5483 (3)	0.3918 (3)	0.6122 (2)	0.0247 (7)	
C3	0.4702 (3)	0.4454 (4)	0.6824 (2)	0.0327 (8)	
H3	0.4934	0.5304	0.7255	0.039*	
C4	0.3580 (3)	0.3667 (4)	0.6845 (3)	0.0374 (9)	
C5	0.3184 (3)	0.2392 (4)	0.6226 (3)	0.0387 (9)	
H5	0.2404	0.1907	0.6280	0.046*	
C6	0.3949 (3)	0.1847 (4)	0.5531 (2)	0.0334 (8)	
H6	0.3711	0.0992	0.5107	0.040*	
C7	0.5087 (3)	0.2634 (3)	0.5496 (2)	0.0251 (7)	
C8	0.6966 (3)	0.3365 (4)	0.5101 (2)	0.0263 (7)	
C9	0.8058 (3)	0.3237 (4)	0.4507 (2)	0.0344 (8)	
H9A	0.8578	0.4190	0.4625	0.041*	
H9B	0.7742	0.3219	0.3801	0.041*	
C10	0.8882 (3)	0.1785 (4)	0.4735 (2)	0.0290 (7)	
C11	0.8469 (4)	0.5118 (4)	0.7462 (3)	0.0505 (11)	
H11A	0.8860	0.5981	0.7871	0.061*	
H11B	0.7840	0.4618	0.7836	0.061*	
C12	0.9460 (4)	0.3907 (5)	0.7297 (3)	0.0584 (12)	
H12A	0.9069	0.2989	0.6959	0.088*	
H12B	0.9889	0.3583	0.7932	0.088*	
H12C	1.0061	0.4369	0.6892	0.088*	

### Atomic displacement parameters (Å^2^)

**Table:**

	*U*^11^	*U*^22^	*U*^33^	*U*^12^	*U*^13^	*U*^23^
S	0.0381 (5)	0.0222 (4)	0.0500 (5)	−0.0057 (4)	−0.0124 (4)	0.0003 (4)
F	0.0489 (13)	0.0634 (15)	0.0605 (14)	0.0130 (12)	0.0265 (11)	0.0092 (12)
O1	0.0296 (12)	0.0246 (11)	0.0286 (12)	0.0014 (10)	−0.0018 (10)	−0.0065 (9)
O2	0.0306 (14)	0.0502 (17)	0.0602 (17)	0.0095 (13)	0.0200 (13)	0.0200 (14)
O3	0.0360 (14)	0.0379 (14)	0.0499 (15)	0.0090 (11)	0.0177 (12)	0.0116 (12)
C1	0.0288 (17)	0.0182 (15)	0.0268 (16)	0.0005 (13)	−0.0051 (13)	0.0020 (12)
C2	0.0282 (16)	0.0197 (15)	0.0245 (16)	0.0045 (13)	−0.0046 (13)	0.0024 (13)
C3	0.041 (2)	0.0254 (17)	0.0309 (18)	0.0074 (15)	0.0003 (16)	0.0013 (14)
C4	0.036 (2)	0.039 (2)	0.040 (2)	0.0126 (16)	0.0109 (17)	0.0143 (16)
C5	0.0277 (18)	0.0320 (19)	0.056 (2)	−0.0019 (15)	0.0017 (17)	0.0157 (17)
C6	0.0317 (19)	0.0238 (16)	0.042 (2)	0.0001 (15)	−0.0079 (16)	0.0053 (15)
C7	0.0270 (17)	0.0192 (15)	0.0278 (17)	0.0021 (13)	−0.0029 (14)	0.0036 (13)
C8	0.0256 (17)	0.0233 (15)	0.0294 (17)	0.0018 (14)	−0.0003 (14)	0.0020 (13)
C9	0.0333 (19)	0.0324 (18)	0.0375 (19)	0.0064 (15)	0.0047 (16)	0.0048 (15)
C10	0.0265 (18)	0.0307 (17)	0.0298 (18)	−0.0012 (15)	0.0032 (14)	−0.0001 (14)
C11	0.062 (3)	0.042 (2)	0.042 (2)	−0.002 (2)	−0.0234 (19)	−0.0088 (18)
C12	0.051 (2)	0.059 (3)	0.060 (3)	0.002 (2)	−0.017 (2)	0.020 (2)

### Geometric parameters (Å, °)

**Table:**

S—C1	1.751 (3)	C5—C6	1.376 (5)
S—C11	1.815 (4)	C5—H5	0.9300
F—C4	1.371 (4)	C6—C7	1.379 (4)
O1—C8	1.379 (4)	C6—H6	0.9300
O1—C7	1.380 (4)	C8—C9	1.477 (4)
O2—C10	1.303 (4)	C9—C10	1.504 (4)
O2—H2	0.85 (5)	C9—H9A	0.9700
O3—C10	1.217 (4)	C9—H9B	0.9700
C1—C8	1.349 (4)	C11—C12	1.491 (5)
C1—C2	1.445 (4)	C11—H11A	0.9700
C2—C3	1.391 (4)	C11—H11B	0.9700
C2—C7	1.395 (4)	C12—H12A	0.9600
C3—C4	1.362 (5)	C12—H12B	0.9600
C3—H3	0.9300	C12—H12C	0.9600
C4—C5	1.385 (5)		
			
C1—S—C11	101.79 (15)	C1—C8—O1	112.0 (3)
C8—O1—C7	105.8 (2)	C1—C8—C9	132.1 (3)
C10—O2—H2	112 (4)	O1—C8—C9	115.9 (3)
C8—C1—C2	106.5 (3)	C8—C9—C10	114.9 (3)
C8—C1—S	125.9 (3)	C8—C9—H9A	108.6
C2—C1—S	127.5 (2)	C10—C9—H9A	108.6
C3—C2—C7	119.4 (3)	C8—C9—H9B	108.6
C3—C2—C1	135.2 (3)	C10—C9—H9B	108.6
C7—C2—C1	105.5 (3)	H9A—C9—H9B	107.5
C4—C3—C2	116.3 (3)	O3—C10—O2	124.3 (3)
C4—C3—H3	121.8	O3—C10—C9	123.5 (3)
C2—C3—H3	121.8	O2—C10—C9	112.2 (3)
C3—C4—F	118.1 (3)	C12—C11—S	114.3 (3)
C3—C4—C5	124.5 (3)	C12—C11—H11A	108.7
F—C4—C5	117.4 (3)	S—C11—H11A	108.7
C6—C5—C4	119.7 (3)	C12—C11—H11B	108.7
C6—C5—H5	120.2	S—C11—H11B	108.7
C4—C5—H5	120.2	H11A—C11—H11B	107.6
C5—C6—C7	116.6 (3)	C11—C12—H12A	109.5
C5—C6—H6	121.7	C11—C12—H12B	109.5
C7—C6—H6	121.7	H12A—C12—H12B	109.5
C6—C7—O1	126.2 (3)	C11—C12—H12C	109.5
C6—C7—C2	123.5 (3)	H12A—C12—H12C	109.5
O1—C7—C2	110.3 (3)	H12B—C12—H12C	109.5
			
C11—S—C1—C8	−101.3 (3)	C8—O1—C7—C2	1.0 (3)
C11—S—C1—C2	83.6 (3)	C3—C2—C7—C6	−0.1 (4)
C8—C1—C2—C3	179.5 (3)	C1—C2—C7—C6	179.3 (3)
S—C1—C2—C3	−4.6 (5)	C3—C2—C7—O1	179.8 (3)
C8—C1—C2—C7	0.4 (3)	C1—C2—C7—O1	−0.8 (3)
S—C1—C2—C7	176.2 (2)	C2—C1—C8—O1	0.2 (3)
C7—C2—C3—C4	−0.2 (4)	S—C1—C8—O1	−175.7 (2)
C1—C2—C3—C4	−179.3 (3)	C2—C1—C8—C9	179.1 (3)
C2—C3—C4—F	−179.1 (3)	S—C1—C8—C9	3.2 (5)
C2—C3—C4—C5	0.2 (5)	C7—O1—C8—C1	−0.7 (3)
C3—C4—C5—C6	0.0 (5)	C7—O1—C8—C9	−179.8 (2)
F—C4—C5—C6	179.3 (3)	C1—C8—C9—C10	109.0 (4)
C4—C5—C6—C7	−0.2 (5)	O1—C8—C9—C10	−72.1 (4)
C5—C6—C7—O1	−179.6 (3)	C8—C9—C10—O3	4.1 (5)
C5—C6—C7—C2	0.3 (4)	C8—C9—C10—O2	−175.1 (3)
C8—O1—C7—C6	−179.1 (3)	C1—S—C11—C12	72.7 (3)

### Hydrogen-bond geometry (Å, °)

**Table:**

*D*—H···*A*	*D*—H	H···*A*	*D*···*A*	*D*—H···*A*
O2—H2···O3^i^	0.85 (5)	1.81 (5)	2.654 (3)	177 (5)

Symmetry codes: (i) −*x*+2, −*y*, −*z*+1.
